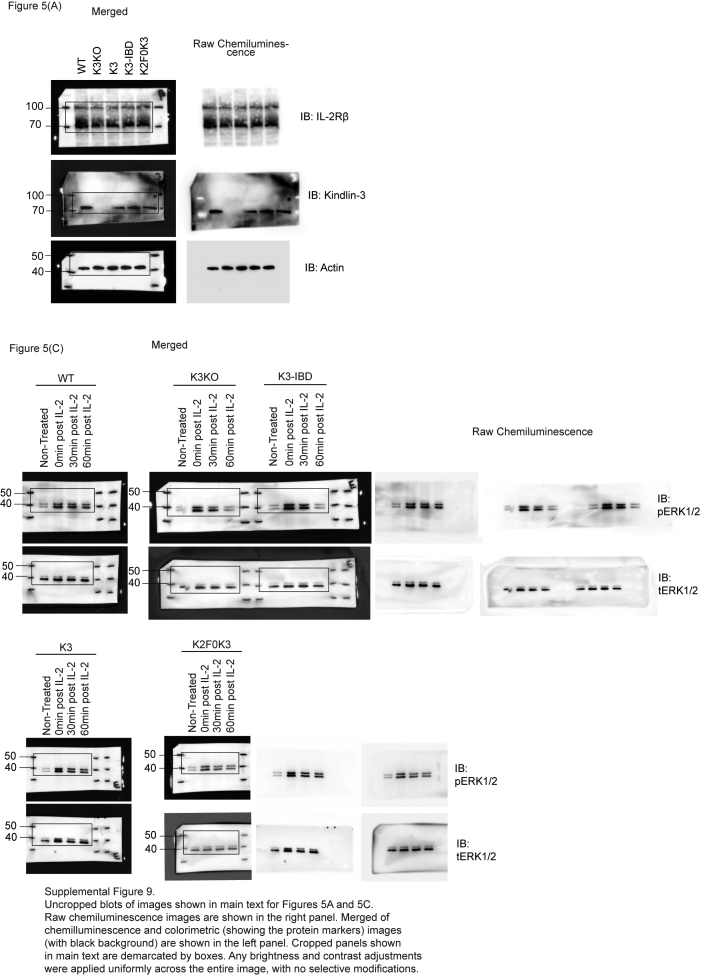# Kindlin-3 sustains cytokine interleukin-2 signaling by linking its receptor to integrin LFA-1

**DOI:** 10.1016/j.jbc.2026.113159

**Published:** 2026-05-15

**Authors:** Zheng-Kai Teng, Neeru Arya, Yinghui Li, Charles Chuah, Surajit Bhattacharjya, Suet-Mien Tan

**Affiliations:** 1 School of Biological Sciences, Nanyang Technological University, Singapore, Singapore; 2 Singapore Immunology Network (SIgN), Agency for Science, Technology and Research, Singapore, Singapore; 3 Department of Haematology, Singapore General Hospital, Singapore, Singapore; 4 Cancer and Stem Cell Biology Program, Duke-NUS Medical School, Singapore, Singapore

**Keywords:** kindlins, cell adhesion, interleukin 2 receptor, integrins, interleukin 2 signaling

## Abstract

Kindlins are adaptor proteins that function synergistically with talin to activate integrins, facilitating cell adhesion and migration. In addition to this role, kindlins have other functions. In immune cells, kindlin-3 is the predominant kindlin paralog. Loss of kindlin-3 due to gene mutations leads to a bleeding disorder and impaired immune function, as observed in patients with leukocyte adhesion deficiency type III (LAD-III). In this study, we demonstrate that kindlin-3 maintains IL-2Rβ at the plasma membrane, a process that relies on the integrin LFA-1. This retention supports IL-2 signaling and helps protect natural killer (NK) cells from apoptosis when IL-2 levels are low. Kindlin-3 also promotes the colocalization of integrin LFA-1 and IL-2Rβ at sites where LFA-1 engages its ligand. Mechanistically, kindlin-3 directly binds the cytoplasmic domain of the interleukin-2 receptor β chain (IL-2Rβ) *via* its F0 subdomain. This interaction specifically targets a membrane-distal region of IL-2Rβ (residues 530–551) with micromolar affinity. Therefore, kindlin-3 acts as a bridging molecule that connects IL-2Rβ to the integrin LFA-1, thereby potentially coordinating cell adhesion with cytokine signaling.

Kindlins are a small family of adaptor proteins that enhance integrin-mediated cell adhesion and migration (1). In humans, three kindlin paralogs exist, each with distinct tissue-specific expression patterns: kindlin-1 is predominantly expressed in the epithelia, kindlin-2 is ubiquitously expressed except for hematopoietic cells, and expression of kindlin-3 is limited to hematopoietic and endothelial cells (2).

Structurally, kindlins contain a 4.1-protein/ezrin/radixin/moesin (FERM) domain composed of F1, F2, and F3 subdomains (3). They also possess an N-terminal F0 domain and a pleckstrin homology (PH) domain embedded within the F2 subdomain (4). Kindlins exhibit a cloverleaf-like conformation in which the F1, F2, and F3 subdomains constitute the central core, whereas the F0 and PH domains project from the F1 and F2 subdomains, respectively (5, 6). Within the F3 subdomain, a phosphotyrosine-binding (PTB) fold specifically recognizes a conserved NPxY/F motif present in the distal C-terminal region of integrin β-subunit cytoplasmic tails (5). Kindlins, together with the cytoskeletal protein talin, enhance integrin ligand-binding affinity (3, 7, 8). Furthermore, kindlins have been reported to facilitate integrin clustering, but the precise mechanism involved remains to be ascertained (5, 9, 10, 11, 12). There is an expanding list of non-integrin binding partners of kindlins (3), suggesting that they have additional functions apart from regulating cell adhesion and migration.

The critical role of kindlin-3 is highlighted by the rare yet severe condition leukocyte adhesion deficiency type III (LAD-III), where mutations in the *FERMT3* gene result in both a bleeding disorder and an impaired immune system (13, 14, 15). In addition to the F3 sub-domain, the F0 sub-domain of kindlin-3 has been shown to disrupt the membrane proximal association between the α and ß cytoplasmic tails in the immune cell integrin LFA-1—a process that is essential for the full activation of LFA-1 (16). Structural analyses show that although the F0 subdomain of kindlin-3 is anchored to the F1/F2/F3 core, it exhibits substantial conformational freedom, unaffected by the oligomeric state of the protein (6). This suggests that the F0 sub-domain of kindlin-3 can have additional binding partners. Indeed, the F0 subdomains of kindlin-3 and kindlin-2 have been reported to interact with paxillin, actin, leupaxin, and Src (3). Whether the F0 subdomain of kindlin-3 has additional binding partners remains to be determined.

IL-2 is a critical cytokine for immune function, directing clonal expansion and differentiation in adaptive immunity (17, 18). Effective IL-2 signaling depends on its engagement with the cell surface IL-2 receptor (IL-2R), a complex comprising α, β, and γ subunits (19). While the dimeric IL-2R (β and γ subunits) is sufficient to mediate signal transduction *via* JAK-STAT pathways (20), the trimeric receptor also includes the α subunit, enhancing ligand affinity up to 100-fold (21). Downstream, IL-2R activation orchestrates MAPK, PI3K, and STAT5 pathways, supporting effector function and immune memory (22, 23, 24, 25, 26, 27). Within the cytoplasmic tail of IL-2Rβ, a membrane-proximal Box1/Box2 region recruits JAK1, more distal tyrosine-containing motifs serve as docking sites for STAT proteins, and a C-terminal degradation/sorting motif regulates receptor endocytosis and downregulation (28). Signal attenuation of IL-2R pathways is tightly regulated through endocytosis. After ligand binding and activation of downstream signaling pathways, the IL-2–IL-2R complex is rapidly internalized. The α-subunit is preferentially recycled (29), whereas the β- and γ-chains, along with the bound IL-2, are targeted for degradation (29, 30).

Natural killer (NK) cells are critical innate lymphocytes that contribute to early defense against infected and transformed cells (31, 32). During an immune response, NK cells are dependent on interleukin-2 (IL-2) for survival, proliferation, and enhanced cytotoxic function (33, 34, 35). Additionally, IL-2 modulates the expression of activating receptors on the NK cell surface (36) and promotes the secretion of effector cytokines such as IFN-γ (37, 38). In addition to promoting cell adhesion and migration, kindlin-3 has been shown to lower the activation threshold of NK cells, although the underlying mechanism remains unclear (39). IL-2 is also critical for T-cell proliferation and function (17, 40). During homotypic T-cell interactions, polarized IL-2/IL-2R–mediated paracrine signaling at the homotypic synapse drives T-cell expansion (41). However, how the IL-2 receptor is recruited to this synapse is not yet understood.

In this study, we demonstrate that kindlin-3 serves as a bridging molecule that physically links the IL-2 receptor to integrin LFA-1. This interaction prolongs the retention of ligand-bound IL-2 receptors on the plasma membrane and enhances cellular responsiveness to IL-2 signaling.

## Results

### Kindlin-3 directly binds IL-2Rβ through specific molecular determinants

**Figure 1:**
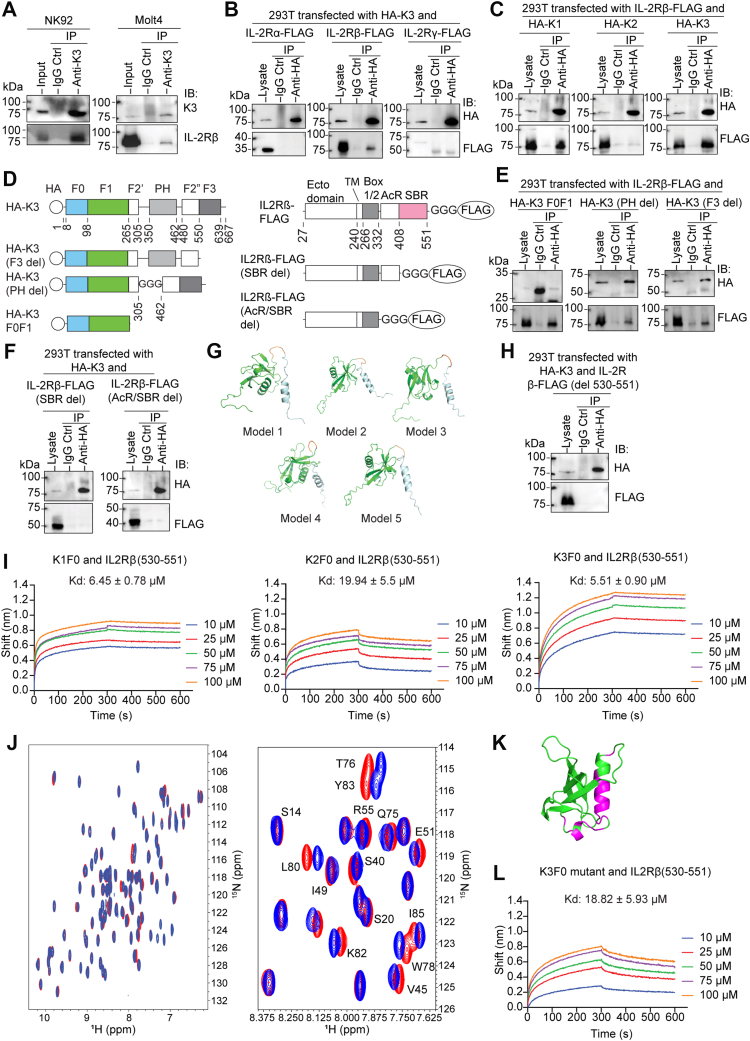
**Kindlin-3 directly binds IL-2Rβ through specific molecular determinants.***A*, endogenous co-immunoprecipitation from NK92 and Molt4 cell lysates. Lysates were immunoprecipitated with anti-kindlin-3 antibody or control IgG and immunoblotted for kindlin-3 and IL-2Rβ. IL-2Rβ is detected at ∼75 kDa in kindlin-3 immunoprecipitates but absent in control IgG precipitates. *B*, receptor subunit specificity analysis. HEK293T cells were co-transfected with N-terminal HA-tagged kindlin-3 (HA-K3) and individual C-terminal FLAG-tagged IL-2 receptor subunits (IL-2Rα-FLAG, IL-2Rβ-FLAG, or IL-2Rγ-FLAG). Lysates were immunoprecipitated with anti-HA antibody or control IgG and immunoblotted for HA and FLAG. Kindlin-3 selectively binds IL-2Rβ but not IL-2Rα or IL-2Rγ. *C*, comparative binding analysis of kindlin family members. HEK293T cells were co-transfected with N-terminal HA-tagged kindlin-1 (HA-K1), kindlin-2 (HA-K2), or kindlin-3 (HA-K3) and C-terminal FLAG-tagged IL-2Rβ (IL-2Rβ-FLAG). Lysates were immunoprecipitated with anti-HA antibody or control IgG and immunoblotted for HA and FLAG. All three kindlin paralogues interact with IL-2Rβ, with kindlin-3 showing the strongest interaction. *D*, schematic representation of kindlin-3 and IL-2Rβ truncation constructs used for domain mapping. Kindlin-3: F0, F1, F2, F3 subdomains and pleckstrin homology (PH) domain. Truncated constructs include HA-K3 (F3 del) lacking the F3 subdomain, HA-K3 (PH del) lacking the PH domain, and HA-K3 (F0F1) containing only F0F1 subdomains. IL-2Rβ: extracellular domain, transmembrane domain (TM), acidic region (AcR), and STAT-binding region (SBR). Truncated constructs include IL-2Rβ-FLAG (SBR del) and IL-2Rβ-FLAG (AcR/SBR del). *E*, domain mapping of kindlin-3. HEK293T cells were co-transfected with N-terminal HA-tagged kindlin-3 truncated constructs (HA-K3 F0F1, HA-K3 PH del, or HA-K3 F3 del) and C-terminal FLAG-tagged IL-2Rβ (IL-2Rβ-FLAG). Lysates were immunoprecipitated with anti-HA antibody and immunoblotted for HA and FLAG. The F0F1 region is sufficient for IL-2Rβ binding; deletion of F3 or PH domains does not affect binding. *F*, domain mapping of IL-2Rβ. HEK293T cells were co-transfected with N-terminal HA-tagged kindlin-3 (HA-K3) and C-terminal FLAG-tagged IL-2Rβ truncated constructs (IL-2Rβ-FLAG SBR del or IL-2Rβ-FLAG AcR/SBR del). Lysates were immunoprecipitated with anti-HA antibody and immunoblotted for HA and FLAG. Deletion of the C-terminal STAT-binding region completely abolishes kindlin-3 binding. *G*, alphaFold 2 Colab was used as an exploratory tool to generate hypothesis regarding the interaction between Kindlin-3 and IL-2Rβ. The five models generated are shown. Kindlin-3 F0 subdomain and IL-2Rβ residues 530 to 551 are highlighted in *green* and *cyan*, respectively. The Gly-linker is highlighted in *orange*. *H*, validation of the Kindlin-3 F0 binding to IL-2Rβ residues 530 to 551. HEK293T cells were co-transfected with N-terminal HA-tagged kindlin-3 (HA-K3) and C-terminal FLAG-tagged IL-2Rβ lacking residues 530 to 551 (IL-2Rβ-FLAG Δ530–551). Lysates were immunoprecipitated with anti-HA antibody and immunoblotted for HA and FLAG. Deletion of residues 530 to 551 completely abolishes kindlin-3 binding. *I*, bio-layer interferometry (BLI) quantification of kindlin F0 domain binding affinities to biotin-tagged IL-2Rβ-derived peptide (residues 530–551). Sensorgrams show association (0–300 s) and dissociation (300–600 s) phases. Data represents mean ± SD from n = 3 biological replicates. *J*, (*left panel*) overlay of ^15^N-^1^H HSQC spectra of K3F0 alone (*red contour*) and in the presence of IL2 derived peptide fragment (*blue contour*). (*Right panel*) A section of ^15^N-^1^H HSQC spectra showing peptide binding induced chemical shift changes of the K3F0 domain. *K*, the two stretches of residues that showed peptide-binding induced chemical shift changes are highlighted in magenta in the K3F0 model. *L*, BLI was performed to measure the binding affinity of biotin-tagged IL-2Rβ-derived peptide and K3F0 mutant carrying substitutions to the corresponding kindlin-2 residues (L46M, I49L, Q52K, I53L, N54D, R55V, and Q75K). Data represent mean ± SD from n = 3 biological replicates.

To investigate whether kindlin-3 interacts with IL-2R, endogenous co-immunoprecipitation (IP) from NK92 and Molt4 cells was performed. Results revealed an interaction between kindlin-3 and IL-2R, with IL-2Rβ as a reporter detected at the expected molecular weight of ∼75 kDa in kindlin-3 immunoprecipitates but absent in control IgG precipitates (Fig. 1*A*). The specificity of kindlin-3 binding to IL-2Rβ was verified by performing co-IP on HEK293T cells that were co-transfected with HA-kindlin-3 and IL-2Rα-FLAG, IL-2Rβ-FLAG or IL-2Rγ-FLAG (Fig. 1*B*). To evaluate the specificity among kindlin paralogs, co-transfection experiments in HEK293T cells revealed that all three kindlin paralogs were capable of interacting with IL-2Rβ, though with differing binding strengths. Among them, kindlin-3 showed the strongest interaction, followed by kindlin-1 and kindlin-2 (Fig. 1*C*).

Kindlin-3 and IL-2Rβ mutant constructs were generated to identify the specific domains involved in their interaction (Fig. 1*D*). Domain mapping revealed that the F0F1 region of kindlin-3 was sufficient for binding to IL-2Rβ, as removal of the F3 subdomain or the PH domain did not disrupt the interaction (Fig. 1*E*). Further truncation of the F0F1 mutant could not be achieved due to low expression levels in the transfected cells. Conversely, deletion of the C-terminal STAT-binding region (SBR) of IL-2Rβ completely abolished its association with kindlin-3, indicating that this region is essential for the interaction (Fig. 1*F*).

**Figure S1:**
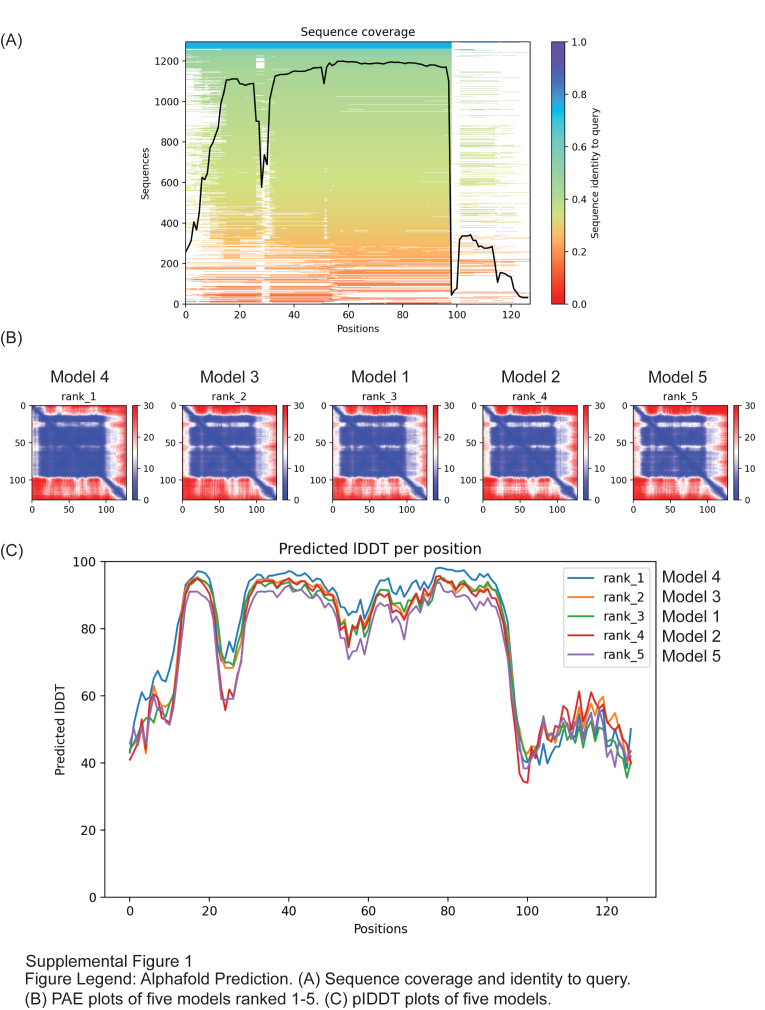


Next, AlphaFold 2 Colab was used as an exploratory tool to generate hypotheses about the interaction between Kindlin-3 F0 subdomain and IL-2Rβ, rather than to predict their binding conformation. Five models were generated based on kindlin-3 F0 linked *via* a Gly-linker to the IL-2Rβ SBR residues 530 to 551 (Figs. 1*G* and S1). Co-IP was performed on HEK293T cells that were co-transfected with HA-Kindlin-3 and IL-2Rβ-FLAG with deletion of residues 530 to 551 (Fig. 1*H*). The deletion effectively abrogated the interaction between kindlin-3 and IL-2Rβ. We then performed bio-layer interferometry (BLI) using recombinantly expressed kindlin F0 domains in bacteria and a synthesized peptide corresponding to IL-2Rβ residues 530 to 551 (Fig. 1*I*). BLI revealed distinct binding affinities among the kindlin paralogs: kindlin-3 (Kd = 5.51 ± 0.90 μM), kindlin-1 (Kd = 6.45 ± 0.78 μM), and kindlin-2 (Kd = 19.94 ± 5.5 μM). These data are consistent with our co-IP data with kindlin-3 F0 domain having approximately 3.6-fold higher affinity to IL-2Rβ residues 530 to 551 as compared to kindlin-2 F0 domain. In addition, the sensogram profile of kindlin-3 F0 binding to the IL-2Rβ peptide shows smooth, concentration-dependent association curves and that can be fit to a 1:1 binding model. However, this model should be interpreted as an operational approximation rather than proof of a single structurally defined binding mode. Given the conformational flexibility of the IL 2Rβ cytoplasmic peptide and the NMR evidence for distributed perturbations, the data support direct micromolar binding but do not define a unique binding mechanism.

We also performed ^15^N-^1^H HSQC nuclear magnetic resonance titration study using ^15^N-labelled kindlin-3 F0 domain and unlabeled peptide corresponding to IL-2Rβ residues 530 to 551 (Fig. 1*J*). The ^15^N-^1^H HSQC spectrum of kindlin-3 F0 was well dispersed in ^1^HN and ^15^N chemical shifts, consistent with a folded protein. HSQC peaks were identified for all non-prolyl residues except for the last three residues (QHR) at the C-terminus and a flexible loop containing residues QKRQ. Chemical shift changes are seen in an overlay of ^15^N-^1^H HSQC spectra of free kindlin-3 F0 (red contour) and kindlin-3 F0 in the presence of the IL-2Rβ peptide (blue contour) (Fig. 1*J*).

**Figure S2:**
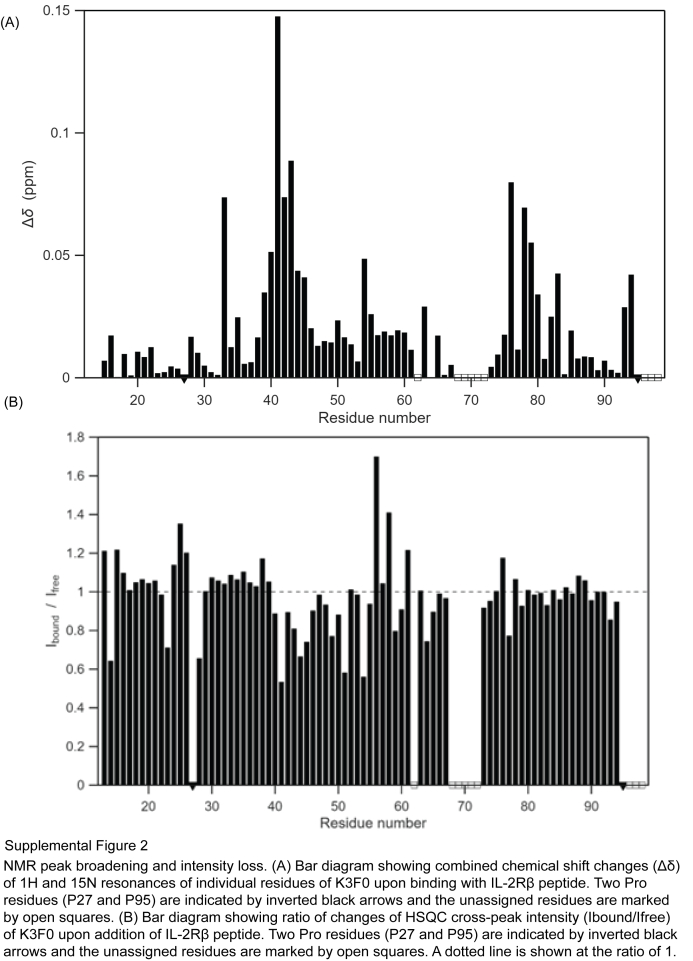


Two regions in kindlin-3 F0 were identified, comprising residues H41, I42, G43, G44, V45, L46, L47, K48, I49, N54 and Q75, T76, W78, T79, L80, K82, Y83, I85 (Fig. S2, *A* and *B*). These residues are highlighted in magenta (Fig. 1*K*). The data are consistent with an interaction between kindlin-3 F0 and the IL-2Rβ peptide. However, HSQC titrations may also reflect conformational changes distal to the binding interface. Therefore, the precise binding surface of kindlin-3 F0 for the IL-2Rβ peptide cannot be defined from these data alone.

Nevertheless, since kindlin-2 F0 exhibits a lower binding affinity for the IL-2Rβ peptide compared to kindlin-3 F0, as determined by BLI, we investigated whether substituting the two regions of kindlin-3 F0 sequence with that from kindlin-2 would reduce its binding affinity. Within these regions, seven residues differ between kindlin-3 and kindlin-2. We therefore generated a kindlin-3 F0 mutant carrying substitutions to the corresponding kindlin-2 residues (L46M, I49L, Q52K, I53L, N54D, R55V, and Q75K). BLI analysis showed that this mutant exhibited reduced binding affinity to the IL-2Rβ peptide compared to wild-type kindlin-3 F0 (Fig. 1*L*), further supporting the interaction between kindlin-3 F0 and IL-2Rβ residues 530 to 551.

### Kindlin-3 retains IL-2Rβ on the cell surface, promoting NK cell survival

**Figure 2:**
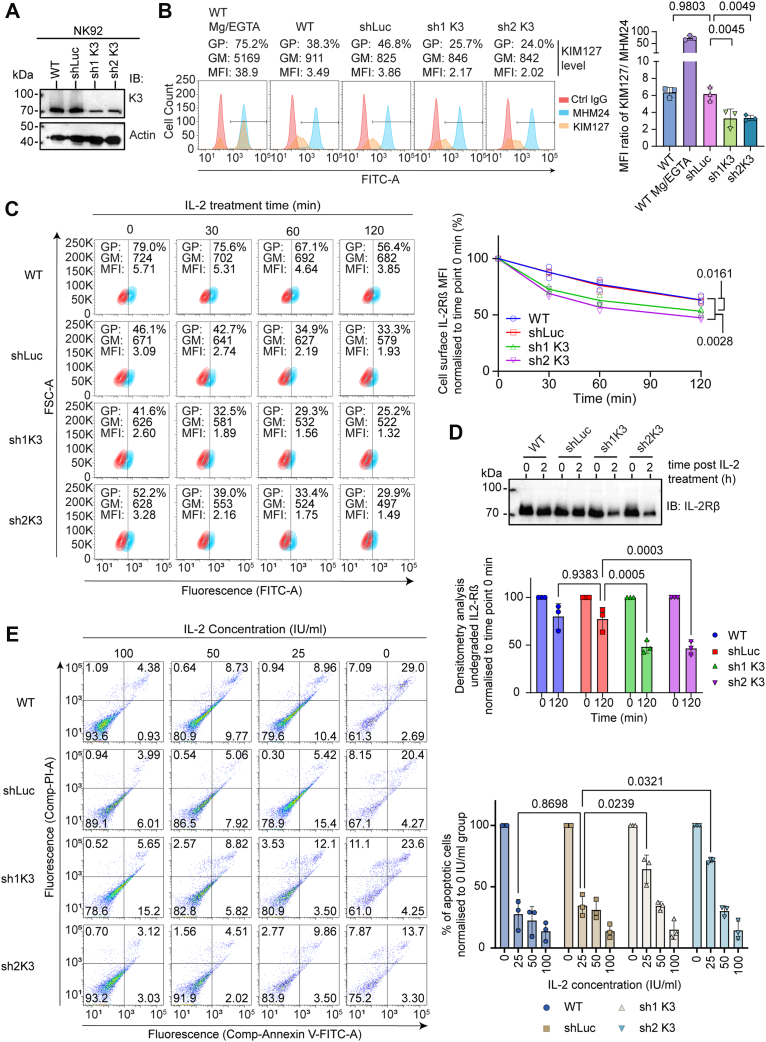
**Kindlin-3 retains IL-2Rβ surface expression and enhances NK cell survival under limiting IL-2 conditions.***A*, validation of kindlin-3 knockdown in NK92 cells. Stable NK92 lines expressing doxycycline-inducible shRNA targeting kindlin-3 (sh1 K3, sh2 K3) or luciferase control (shLuc) were treated with 1 μg/ml doxycycline for 5 days. Immunoblot analysis confirmed >50% reduction in kindlin-3 protein levels (∼75 kDa) in knockdown lines compared to control. β-Actin served as loading control. *B*, LFA-1 activation status in kindlin-3-depleted NK92 cells. Cells were analyzed by flow cytometry using conformation-specific antibody KIM127 (recognizing active LFA-1) and anti-LFA-1 antibody MHM24 (total LFA-1). LFA-1 activation is expressed as KIM127/MHM24 mean fluorescence intensity (MFI) ratio. Mg^2+^/EGTA treatment (5 mM MgCl_2_/1.5 mM EGTA) served as positive control for maximal LFA-1 activation. Data represents mean ± SD from n = 3 biological replicates. One-way ANOVA followed by Dunnett's multiple comparisons test. *C*, kindlin-3 depletion accelerates IL-2Rβ internalization in NK92 cells. Cells were IL-2-starved, then stimulated with 100 IU/ml IL-2 for the indicated times, and surface IL-2Rβ levels quantified by flow cytometry using anti-CD122 antibody. Plot on the right shows data with SD from n = 3 biological replicates. Two-way ANOVA followed by Dunnett's multiple comparisons test. *D*, biochemical validation of IL-2Rβ surface loss in kindlin-3-depleted cells. Cell surface proteins were biotinylated at 4 °C before IL-2 stimulation (100 IU/ml, 120 min at 37 °C), then isolated by streptavidin pulldown and analyzed by immunoblotting for IL-2Rβ. Representative immunoblots (*top*) and densitometric quantification normalized to t = 0 (*bottom*). Data represents mean ± SD from n = 3 biological replicates. Two-way ANOVA followed by Dunnett's multiple comparisons test. *E*, kindlin-3 depletion impairs NK92 survival at limiting IL-2 concentrations. Cells were IL-2-starved for 24 h, then cultured with indicated IL-2 doses (0, 25, 50, or 100 IU/ml) for 48 h before Annexin V/propidium iodide staining and flow cytometry analysis. Apoptotic cells were quantified and expressed as percentage of IL-2-starved (0 IU/ml) controls. Data represents mean ± SD from n = 3 biological replicates. Two-way ANOVA followed by Dunnett's multiple comparisons test.

NK-92 cells are IL-2-dependent for survival and proliferation in culture, making them an excellent model for investigating IL-2 receptor regulation. To assess the role of kindlin-3 in regulating IL-2 receptor signaling, stable NK92 cells with doxycycline-inducible knockdown of kindlin-3 (sh1 K3 and sh2 K3) or a control construct (shLuc) were established. After 5 days of doxycycline treatment, immunoblot analysis verified a reduction of more than 50% in kindlin-3 protein levels (Fig. 2*A*). Given that kindlin-3 positively regulates integrin activity (42), we also confirmed that silencing kindlin-3 expression effectively reduces integrin LFA-1 activation based on flow cytometry using the conformation-specific monoclonal antibody KIM127 that reports an extended and activated integrin LFA-1 (Fig. 2*B*). Exogenous activating agents, Mg/EGTA were used as a positive control.

We then performed flow cytometry analysis to examine IL-2 receptor dynamics in kindlin-3 depleted cells. When treated with IL-2, kindlin-3-depleted cells showed accelerated IL-2Rβ internalization relative to wild-type cells and shLuc control cells (Fig. 2*C*). Time course analysis revealed progressive receptor loss in kindlin-3-depleted cells, with differences apparent as early as 30 min post-IL-2 treatment. To confirm this observation, we employed an alternative method in which cell surface proteins were biotinylated, and IL-2Rβ levels were subsequently monitored over time by immunoblotting (Fig. 2*D*). After 120 min of IL-2 stimulation, wild-type NK92 cells retained substantial surface IL-2Rβ, comparable to shLuc controls. In contrast, both knockdown lines exhibited a significant reduction in protein levels. The biotinylation data closely matched the flow cytometry results, indicating that the decreased detection of surface IL-2Rβ reflects actual protein loss *via* internalization rather than changes in antibody accessibility in the flow cytometry analysis. This data also suggests that kindlin-3 retains the IL-2R on the cell surface in the presence of IL-2.

**Figure S3:**
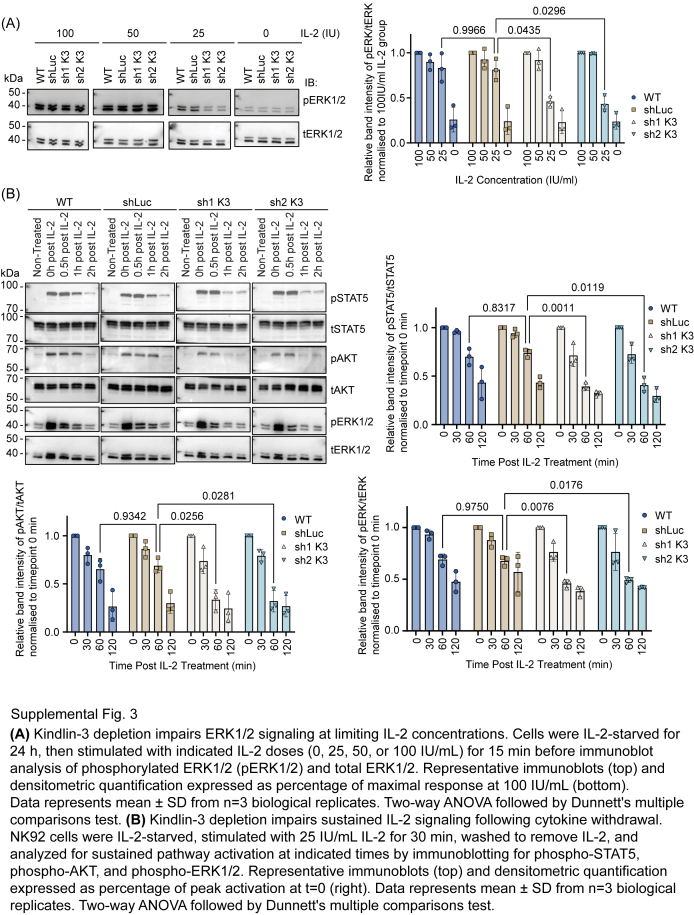


Next, we evaluated how kindlin-3 depletion affects the survival of NK92 cells under conditions of progressively reduced IL-2 concentrations by performing flow cytometry-based apoptosis assay (Fig. 2*E*). When cells were treated with either 100 or 50 IU/ml, there is no significant difference between all groups of cells. However, at 25 IU/ml, kindlin-3-depleted cells exhibited marked apoptosis as compared to wild-type and shLuc control cells. Downstream signaling analysis revealed that kindlin-3–depleted cells exhibit impaired ERK1/2 phosphorylation under limiting IL-2 conditions (Fig. S3*A*). At 25 IU/ml IL-2, control cells maintained robust ERK1/2 activation, whereas kindlin-3 knockdown cells showed significantly reduced activation. Taken together, these data demonstrate that kindlin-3–deficient cells displayed both diminished ERK1/2 signaling and increased apoptosis when IL-2 levels were low, indicating that kindlin-3 is essential for ERK1/2–mediated survival under suboptimal IL-2 conditions.

In addition to the ERK1/2 signaling pathway, IL-2 binding to IL-2R also activates STAT5 and AKT (17). Therefore, we performed IL-2 transient stimulation followed by washout experiments to assess the impact of kindlin-3 depletion on all three signaling pathways (Fig. S3*B*). In kindlin-3–depleted cells, activation of all three signaling pathways was reduced 1 h after IL-2 treatment compared to wild-type and shLuc control cells. These data confirm that kindlin-3 positively regulates and sustains IL-2R downstream signaling.

### IL-2Rβ retention requires integrin-dependent Kindlin-3 scaffolding

**Figure 3:**
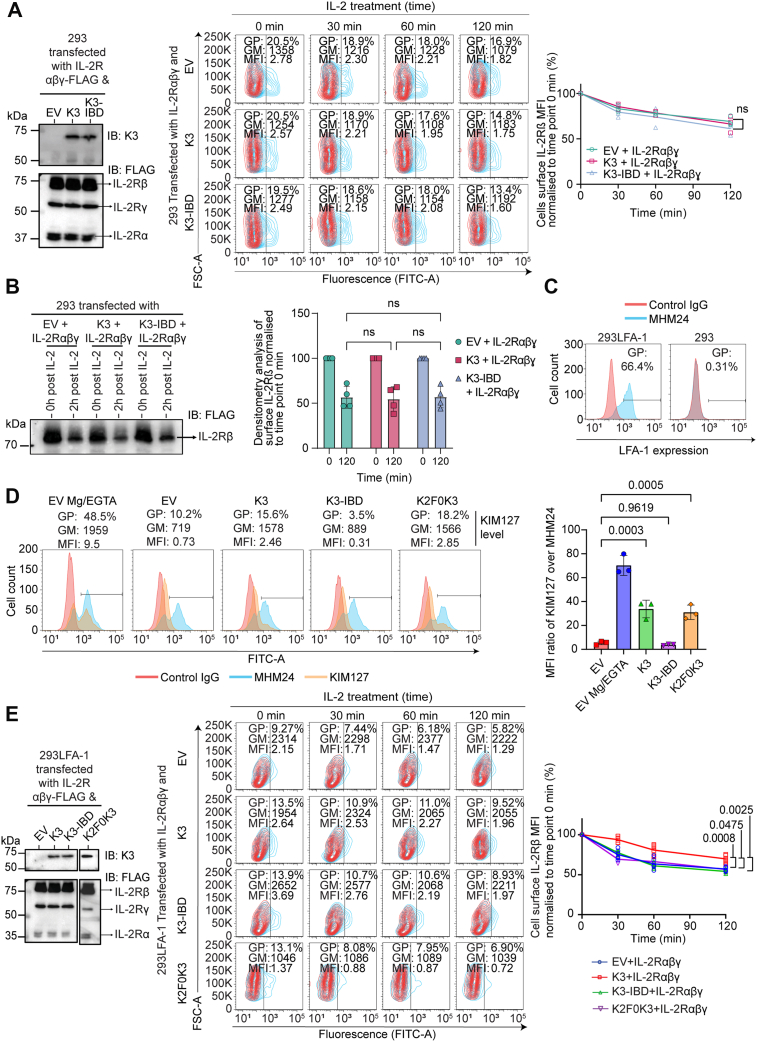
**Integrin-dependent IL-2Rβ stabilization by kindlin-3.***A*, IL-2Rβ internalization in integrin-deficient HEK293 cells. Cells were co-transfected with FLAG-tagged IL-2 receptor chains (IL-2Rα-FLAG, IL-2Rβ-FLAG, IL-2Rγ-FLAG) along with HA-tagged wild-type kindlin-3 (HA-K3), HA-K3 IBD (integrin-binding defective mutant), or empty vector (EV) control. Construct expression was validated by immunoblot analysis. Cells were stimulated with 100 IU/ml IL-2 for the indicated times and surface IL-2Rβ levels quantified by flow cytometry using anti-CD122 antibody. All three conditions showed comparable receptor internalization kinetics, demonstrating that kindlin-3 expression alone cannot stabilize IL-2Rβ in the absence of integrin partners. Data represents mean ± SD from n = 3 biological replicates. *B*, biochemical validation showing kindlin-3 cannot stabilize IL-2Rβ in integrin-deficient cells. Parental HEK293 cells were transfected with kindlin-3 constructs and IL-2 receptors. Surface proteins were biotinylated at 4 °C, cells were stimulated with 100 IU/ml IL-2 for 120 min at 37 °C, and biotinylated proteins isolated by streptavidin pulldown followed by immunoblot analysis for IL-2Rβ. Data represents mean ± SD from n = 4 biological replicates. Two-way ANOVA; no significant differences. *C*, LFA-1 (αLβ2 integrin) expression validation in HEK293-LFA-1 cells. Flow cytometry analysis confirmed stable expression of LFA-1 compared to parental HEK293 cells using MHM24 antibody. *D*, kindlin-3-mediated LFA-1 activation requires the F3 subdomain but not F0 domain identity. HEK293-LFA-1 cells were transfected with empty vector (EV), wild-type HA-K3, HA-K3 IBD, or K2F0K3 chimera (kindlin-3 with F0 subdomain replaced by kindlin-2 F0). LFA-1 activation was measured by flow cytometry using conformation-specific antibody KIM127 (recognizing active extended conformation), normalized to total LFA-1 (MHM24 antibody), and expressed as KIM127/MHM24 MFI ratio. Mg^2+^/EGTA treatment (5 mM MgCl_2_/1.5 mM EGTA) served as positive control for maximal LFA-1 activation. Data represents mean ± SD from n = 3 biological replicates. One-way ANOVA followed by Dunnett's multiple comparisons test. ns, not significant. *E*, IL-2Rβ internalization in integrin-expressing HEK293-LFA-1 cells requires both integrin engagement and IL-2Rβ binding. Cells were co-transfected with IL-2 receptor chains and kindlin-3 constructs (wild-type HA-K3, HA-K3 IBD, K2F0K3, or empty vector), then stimulated with 100 IU/ml IL-2 for 120 min and surface IL-2Rβ quantified by flow cytometry. Data represents mean ± SD from n = 6 (EV & K3) and n = 3 (K3-IBD & K2F0K3) biological replicates. Two-way ANOVA followed by Dunnett's multiple comparisons test.

We hypothesize that kindlin-3 retains IL-2R on the plasma membrane by binding to integrins, thereby acting as a bridging molecule that tethers IL-2R to integrins. To investigate this, we first performed co-transfection experiments using wild-type HEK293 cells, which lack endogenous integrin LFA-1. HEK293 cells were co-transfected with all three IL-2R subunits along with either empty vector (EV), wild-type kindlin-3, or kindlin-3 that is integrin-binding defective (IBD). Cells were then assessed for IL-2R internalization upon IL-2 treatment using flow cytometry (Fig. 3*A*). No significant differences were observed among the different groups of transfected cells for the entire 2 h duration. This was also confirmed by cell surface biotinylation assay (Fig. 3*B*)

Next, we generated a stable HEK293 cell line expressing integrin LFA-1, which was confirmed by flow cytometry analysis (Fig. 3*C*). We assessed the impact of expressing either wild-type kindlin-3 or its mutants on LFA-1 activity in these cells using the reporter monoclonal antibody KIM127 *via* flow cytometry (Fig. 3*D*). The results show that LFA-1 was activated in cells expressing either wild-type kindlin-3 or the kindlin-3 chimera (K2F0K3), in which the F0 domain of kindlin-3 is replaced with that of kindlin-2, compared with cells expressing kindlin-3 (IBD) or an EV. Mg/EGTA treatment was included as a positive control in this experiment. These data confirmed that kindlin-3 can activate LFA-1 in these cells, and this is disrupted by mutations of the integrin-binding site in the F3 domain of kindlin-3.

**Figure S4:**
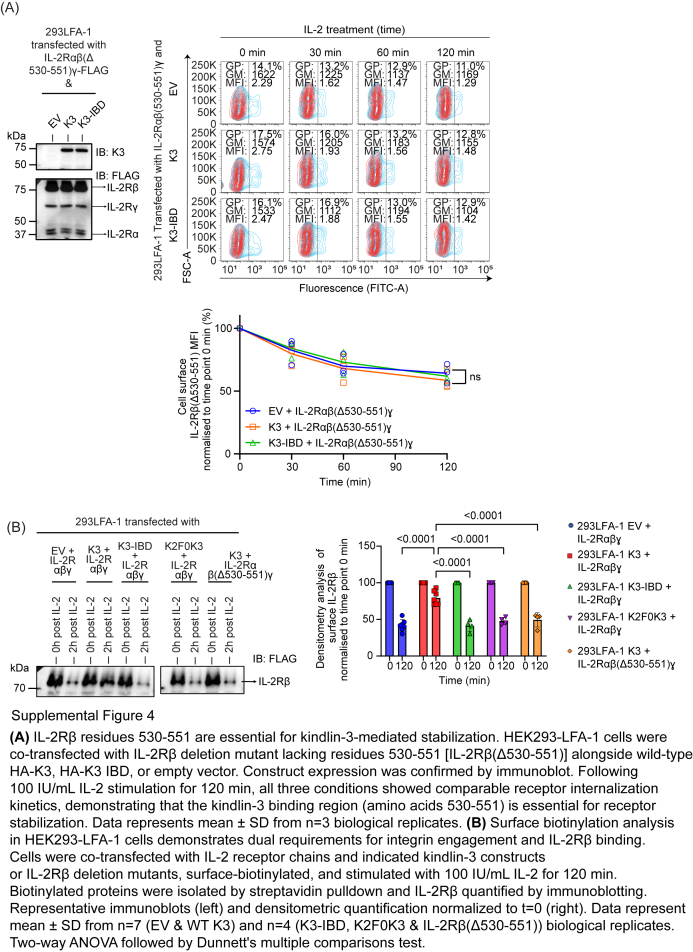


To investigate whether LFA-1 is required for kindlin-3–mediated retention of IL-2R on the plasma membrane, HEK293-LFA-1 cells were co-transfected with all three IL-2R subunits along with either an empty vector (EV), wild-type kindlin-3, kindlin-3 (IBD), or K2F0K3. IL-2R internalization following IL-2 treatment was then analyzed by flow cytometry (Fig. 3*E*). Cells expressing wild-type kindlin-3 exhibited the highest levels of IL-2R retention on the plasma membrane compared with all other groups. These results indicate that both the integrin-binding ability of kindlin-3 and its capacity to interact with IL-2Rβ *via* the F0 domain are necessary for maintaining IL-2Rβ on the plasma membrane in the presence of IL-2 stimulation. We also conducted similar experiments using a mutant IL-2Rβ in which residues 530 to 551 were deleted (Fig. S4*A*). No differences were observed among the different groups in terms of IL-2R retention on the plasma membrane, consistent with the critical role of the 530 to 551 region in mediating the interaction between IL-2Rβ and kindlin-3. Similar observations were made when we performed the cell surface biotinylation assay (Fig. S4*B*).

### Kindlin-3 extends the duration of IL-2 signaling in an integrin-dependent manner

**Figure 4:**
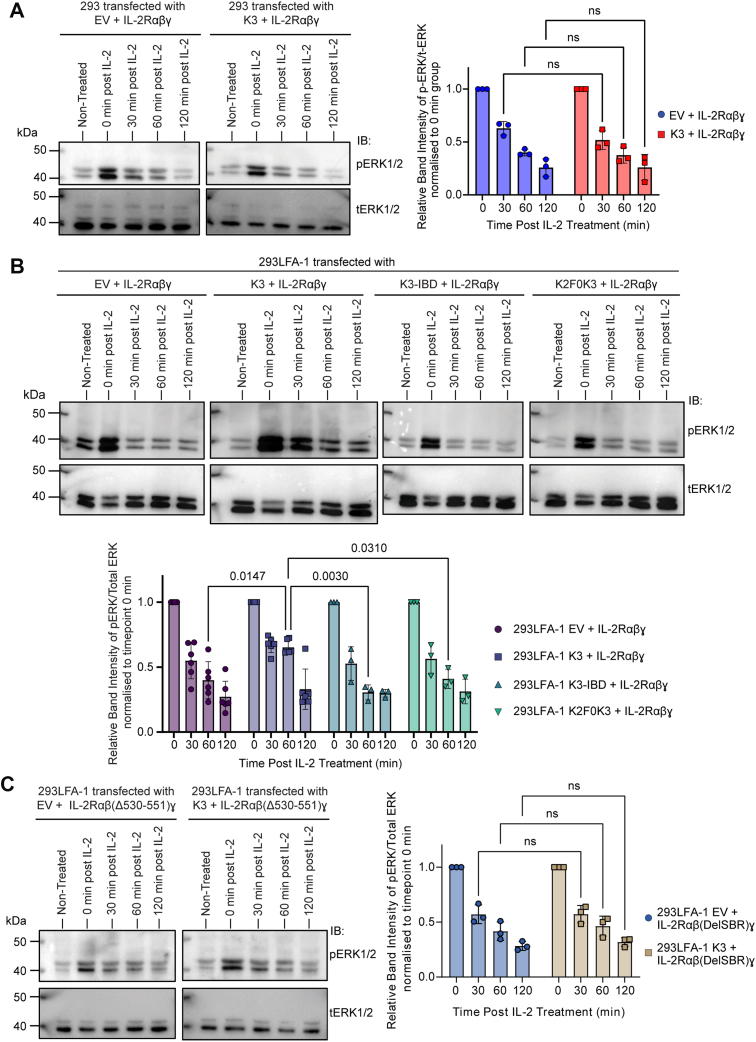
**Kindlin-3 prolongs IL-2 signaling in an integrin-dependent manner.***A*, ERK1/2 signaling kinetics in integrin-deficient HEK293 cells. Cells were co-transfected with IL-2 receptor chains (IL-2Rα, IL-2Rβ, IL-2Rγ) and either empty vector (EV) or HA-kindlin-3, then stimulated with 100 IU/ml IL-2 for 30 min, washed to remove IL-2, and analyzed for sustained ERK1/2 phosphorylation at the indicated times post-withdrawal by immunoblotting. Representative immunoblots (*left*) show phospho-ERK1/2 (pERK1/2) and total ERK1/2 (tERK1/2). Densitometric quantification expressed as pERK1/2/tERK1/2 ratio normalized to t = 0 (*right*). Both EV and kindlin-3 conditions showed similar pERK1/2 patterns: elevated at 0 to 30 min post-withdrawal, returning to baseline by 60 min. Data represent mean ± SD from n = 3 biological replicates. Two-way ANOVA; no significant differences between conditions. *B*, ERK1/2 signaling persistence in integrin-expressing HEK293-LFA-1 cells requires both integrin engagement and IL-2Rβ binding. Cells were co-transfected with IL-2 receptors and kindlin-3 constructs [wild-type (WT) HA-K3, HA-K3 IBD (integrin-binding defective), K2F0K3 chimera, or empty vector], treated with 100 IU/ml IL-2 for 30 min, washed, and analyzed for sustained ERK1/2 phosphorylation over time. Representative immunoblots (*top*) and quantification (bottom) showing pERK1/2/tERK1/2 ratios normalized to t = 0. Data represents mean ± SD from n = 6 (EV & WT K3) and n = 3 (K3-IBD & K2F0K3) biological replicates. Two-way ANOVA followed by Dunnett's multiple comparisons test. *C*, IL-2Rβ residues 530 to 551 are required for kindlin-3-mediated signaling persistence. HEK293-LFA-1 cells were co-transfected with IL-2Rβ deletion mutant [IL-2Rβ(Δ530–551)] lacking the kindlin-3 binding region, alongside empty vector or wild-type HA-kindlin-3. Following 100 IU/ml IL-2 treatment for 30 min and washout, sustained ERK1/2 phosphorylation was monitored by immunoblotting. Representative immunoblots (*left*) and quantification (*right*). Both conditions showed similar pERK1/2 decay kinetics, with signal returning to baseline by 60 min, demonstrating that IL-2Rβ residues 530 to 551 are essential for kindlin-3-mediated signaling persistence. Data represents mean ± SD from n = 3 biological replicates. Two-way ANOVA followed by Dunnett's multiple comparisons test. ns, not significant.

Since kindlin-3–mediated retention of IL-2R on the plasma membrane requires LFA-1, we hypothesized that this dependence on LFA-1 modulates the temporal kinetics of IL-2R-induced ERK1/2 activation. To this end, we performed a transient stimulation and washout assay (Fig. 4*A*). HEK293 cells were transfected with all three subunits of the IL-2 receptor together with either an empty vector (EV) or kindlin-3. The cells were stimulated with IL-2 for 30 min, followed by a washout. ERK1/2 phosphorylation levels were then assessed by immunoblotting at different time points. There was no difference in the kinetics of ERK1/2 phosphorylation between the two groups of cells. Next, we conducted a similar assay using a stable HEK293 cell line expressing LFA-1 (Fig. 4*B*). The cells were transfected with all three IL-2R subunits together with either an empty vector (EV), kindlin-3, kindlin-3 (IBD), or K2F0K3. Compared with the other groups, cells expressing kindlin-3 exhibited sustained elevation of ERK1/2 phosphorylation for up to 1 h. This effect is specific to IL-2R signaling rather than a nonspecific consequence of kindlin-3 ectopic expression, as deletion of residues 530 to 551 in IL-2Rβ effectively abolished sustained ERK1/2 phosphorylation (Fig. 4*C*). Collectively, these data demonstrate that the retention of IL-2R on the plasma membrane by kindlin-3 depends on LFA-1, leading to prolonged activation of ERK1/2 signaling.

### Kindlin-3 functions as a bridging molecule that recruits IL-2R to sites of LFA-1 contact

**Figure 5:**
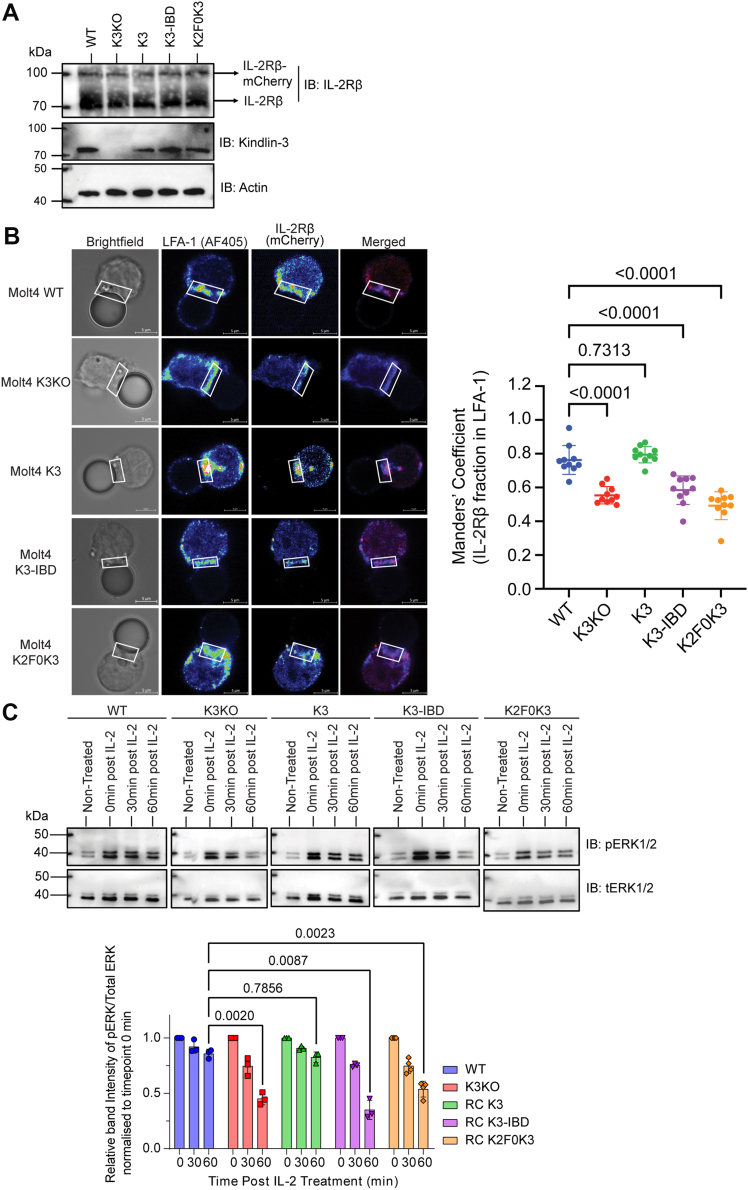
**Kindlin-3 organizes LFA-1/IL-2Rβ spatial microdomains at sites of integrin engagement.***A*, generation and validation of Molt4 cell lines expressing kindlin-3 variants. CRISPR-Cas9-generated kindlin-3 knockout (K3KO) Molt4 cells were reconstituted with wild-type kindlin-3 (K3), integrin-binding defective mutant (K3 IBD), or K2F0K3 chimera (kindlin-3 with F0 subdomain replaced by kindlin-2 F0). Wild-type (WT) Molt4 cells served as control. All lines were transduced with IL-2Rβ-mCherry for fluorescence detection. *Top*: immunoblot validation showing kindlin-3 expression (∼75 kDa) in WT and reconstituted lines, absence in K3KO, and β-actin loading control. *Bottom*: IL-2Rβ-mCherry expression confirmed by immunoblotting with anti-IL-2Rβ antibody (∼100 kDa for IL-2Rβ-mCherry fusion). *B*, LFA-1 and IL-2Rβ colocalization at ICAM-1 engagement sites. Molt4 cells were incubated with ICAM-1-coated 8 μm polystyrene beads in the presence of Mg^2+^/EGTA to activate integrins, then plated on poly-L-lysine-coated dishes and analyzed by confocal microscopy. *Left*: representative confocal images showing LFA-1 (Alexa Fluor 405, *blue*) and IL-2Rβ-mCherry (*red*) distribution at cell-bead contact sites. *Right*: Quantification of colocalization between IL-2Rβ (mCherry fluorescence) and LFA-1 (Alexa Fluor 405 fluorescence) at bead contact sites was performed using Manders’ coefficient. All mutant/KO conditions showed significantly reduced colocalization compared to WT (*p* < 0.0001). Data represent mean ± SD from n = 10 biological replicates. One-way ANOVA followed by Dunnett's multiple comparisons test. Scale bar: 5 μm. *C*, sustained ERK1/2 signaling in Molt4 cells requires both kindlin-3 integrin-engagement and IL-2Rß binding domains. Cells were serum starved overnight and stimulated with 100 IU/ml IL-2 for 30 min, washed, and analyzed for sustained ERK1/2 phosphorylation over time. Representative immunoblots (*top*) and quantification (*bottom*) showing pERK1/2/tERK1/2 ratios normalized to t = 0. Data represents mean ± SD from n = 3 (WT, K3KO, K3, K3-IBD) and n = 4 (K2F0K3) biological replicates. Statistical significance was determined by two-way ANOVA followed by Dunnett’s multiple comparisons test.

Previous study has shown that IL-2R localizes to LFA-1-enriched immune synapse, but the mechanism remains unknown (41). Our findings demonstrate that kindlin-3 plays an important role in this process. To visualize the recruitment of IL-2R to LFA-1 contact sites mediated by kindlin-3, we generated a stable MOLT-4 T cell line ectopically expressing IL-2Rβ fused to a C-terminal mCherry tag (Fig. 5*A*). We then performed CRISPR-Cas9n–mediated knockout of kindlin-3 in this cell line, followed by reconstitution with either wild-type kindlin-3, kindlin-3 (IBD), or K2F0K3. We did not use NK92 cells for this experiment due to poor viability following kindlin-3 knockout.

Next, the cells were incubated with ICAM-1–coated polystyrene beads in the presence of Mg/EGTA to induce LFA-1 activation, thereby bypassing the requirement for kindlin-3. The cell-bead doublets were stained with anti-LFA-1 antibody and examined under confocal laser scanning microscopy (Fig. 5*B*). IL-2Rβ was effectively recruited to LFA-1-ICAM-1 contact sites in wild-type and kindlin-3 reconstituted cells as compared to others, demonstrating that kindlin-3 is required for localizing IL-2Rβ to the immune synapse.

In addition, we assessed whether ERK1/2 phosphorylation following IL-2 treatment is sustained in these cells (Fig. 5*C*). Sustained ERK1/2 phosphorylation was observed in wild-type MOLT-4 and kindlin-3–reconstituted cells, whereas it was reduced in kindlin-3 knockout cells, including those reconstituted with kindlin-3 (IBD) or K2F0K3. These findings are consistent with similar experiments in HEK293 transfected cells described earlier.

## Discussion

Our finding that kindlin-3 directly binds IL-2Rβ and regulates IL-2 receptor signaling reveals a new mechanism integrating integrin-mediated adhesion with cytokine signaling. While kindlin-3 is known as an essential integrin co-activator in hematopoietic cells, its ability to also engage IL-2R broadens its functional scope. The F0 subdomain of kindlin-3 binds to the membrane-distal region of IL-2Rβ with a dissociation constant of ∼ 6 μM, thereby placing this regulatory mechanism within the physiological range typical of dynamic signaling (43). Nevertheless, this interaction is stronger than the binding of kindlin-3 to the integrin β1 tail (Kd ∼200 μM) (6), as well as the interactions of the talin F3 subdomain with integrin cytoplasmic tails (∼490 μM for β1A and ∼273 μM for β3) (44). While talin and kindlin interactions with integrin tails are relatively weak and cooperative in nature, the stronger kindlin-3 F0–IL-2Rβ interaction is likely to support more sustained and specific signaling regulation.

It should also be noted that kindlin-1 shows a similar dissociation constant for IL-2Rβ, whereas kindlin-2 binds more weakly, indicating that F0 domain-mediated cytokine receptor binding is a function selectively conserved in kindlin-1 and kindlin-3, rather than a universal feature of all kindlins. The co-expression of kindlin-1 and IL-2R in intestinal epithelial cells (45, 46, 47), suggests that although this molecular interaction operates in parallel, it serves distinct biological roles. In hematopoietic cells, kindlin-3 links integrin engagement to IL-2R signaling, coordinating cell adhesion with proliferation and survival in immune cells such as T and NK cells. By contrast, in intestinal epithelial cells, kindlin-1 may help retain IL-2R on the plasma membrane to regulate barrier integrity (48, 49) and maintain epithelial homeostasis (50). Receptor stabilization also appears to be a conserved functional feature across the kindlin family, although the mechanisms involved differ between paralogs. Kindlin-1 and kindlin-2 both stabilize EGFR through direct, integrin-independent interactions that prevent receptor degradation, with kindlin-1 blocking lysosomal-mediated degradation (51) and kindlin-2 preventing ubiquitination (52). In contrast, our findings demonstrate that kindlin-3-mediated IL-2Rβ stabilization operates through an integrin-dependent mechanism requiring formation of a tripartite LFA-1-kindlin-3-IL-2Rβ complex, where both integrin engagement and receptor binding are obligate components.

The integrin-dependent retention mechanism described here extends beyond IL-2 signaling due to the shared use of receptor components by IL-15. Both IL-2 and IL-15 utilize a common heterodimeric receptor complex composed of a β-subunit (IL-2/15Rβ) and a γ chain (γc) for signal transduction (53), while each cytokine’s unique α-subunit (IL-2Rα for IL-2, IL-15Rα for IL-15) confers high affinity binding of the cytokine to the signaling receptor complex (54, 55). This receptor sharing underlies the overlapping downstream signaling pathways (56, 57) and consequent biological functions (58, 59). Physiologically, IL-15 plays an essential role in peripheral NK cell homeostasis by promoting anti-apoptotic signaling (60). Unlike IL-2, IL-15 is primarily trans-presented by IL-15Rα-expressing cells, such as dendritic cells, in specific tissue niches (61), generating regulated local microenvironments that fine-tune NK cell maintenance (62). The role of kindlin-3 in retaining IL-2/15Rβ on the plasma membrane is thus critical to amplify NK cell responsiveness under limiting cytokine conditions, enabling peripheral NK cells to sustain viability and functional competence.

The kindlin-3-mediated bridging of LFA-1 and IL-2Rβ provides a molecular explanation for the previously reported localization of IL-2R at homotypic T-cell synapse (41). LFA-1 clustering at cell-cell contact points recruits kindlin-3, which captures and polarizes IL-2Rβ to the synaptic interface. This creates organized signaling microdomains where IL-2R is enriched precisely where paracrine cytokine delivery occurs. This adaptor-mediated spatial organization resembles integrin-associated scaffolds in focal adhesions. Tensin, for instance, bridges integrin cytoplasmic tails to the actin cytoskeleton through its dual binding domains (63). However, while focal adhesion adaptors like tensin link adhesion machinery to cytoskeletal elements for mechanotransduction, kindlin-3 represents a distinct functional paradigm. It couples cell-cell adhesion to cytokine signaling, thereby integrating spatial cues from integrin engagement with recruitment of cytokine receptors to sites of enriched cytokine delivery. This mechanism reveals that integrin-associated adaptors can orchestrate functions beyond adhesion and mechanotransduction, extending to the regulation of cytokine receptor positioning and signaling capacity.

From a therapeutic perspective, the kindlin-3/IL-2 receptor axis presents opportunities for pharmacological intervention that exploit receptor retention as a tunable parameter for modulating immune responses. In autoimmune diseases and transplant rejection, disrupting the kindlin-3-IL-2Rβ interaction could reduce IL-2 receptor retention and downstream signaling without completely ablating cytokine responsiveness, potentially offering a more nuanced approach than current immunosuppressive strategies that broadly inhibit IL-2 production or receptor function (64, 65). This partial dampening of IL-2 signaling may preserve basal immune surveillance while attenuating pathogenic T cell expansion.

Conversely, in cancer immunotherapy, enhancing kindlin-3-mediated IL-2Rβ stabilization represents a compelling strategy to improve the persistence and effector function of engineered immune cells operating in cytokine-depleted tumor microenvironments. CAR-T and CAR-NK cell therapies have achieved remarkable success in hematological malignancies but exhibit limited efficacy in solid tumors, where poor expansion, inadequate persistence, and functional exhaustion remain critical obstacles (66). The immunosuppressive tumor microenvironment is characterized by cytokine limitation and metabolic stress, causing CAR-T cells to undergo activation-induced cell death and lose functional capacity (67). Current strategies to overcome these barriers include armoring CAR-T cells with transgenic cytokine expression or constitutively active cytokine receptors (68), but systemic cytokine administration risks severe toxicities, and autonomous cytokine secretion can trigger cytokine release syndrome (69). Enhancing kindlin-3-mediated IL-2R stabilization offers a mechanistically distinct alternative that amplifies endogenous IL-2 signaling without increasing cytokine production. By prolonging IL-2R surface retention, even scarce autocrine or paracrine IL-2 within the tumor microenvironment could generate sustained STAT5, AKT, and ERK signaling sufficient to maintain CAR cell metabolic fitness, resist apoptosis, and support memory differentiation. This approach could be implemented through genetic engineering strategies by expressing stabilized kindlin-3 variants with enhanced F0 domain binding or co-expressing molecular adaptors that reinforce the LFA-1-kindlin-3-IL-2Rβ bridging complex. Such receptor-stabilization approaches would be particularly synergistic with tumor-reactive CAR cells that naturally cluster LFA-1 at immunological synapses, precisely where kindlin-3-mediated IL-2R enrichment would amplify responsiveness to locally concentrated cytokines. Importantly, this strategy would selectively enhance signaling in activated, tumor-engaged T cells while avoiding systemic toxicity associated with constitutive cytokine expression or administration.

## Experimental procedures

### Chemicals and reagents

All standard laboratory chemicals and reagents were of analytical grade and were purchased from Bio-Rad Laboratories (Hercules, CA), Merck, or Sigma-Aldrich (St Louis, MO) unless otherwise specified.

### Plasmid constructs

All plasmid constructs were generated using standard molecular biology techniques. Target genes were amplified by PCR with gene-specific primers containing appropriate restriction enzyme sites. PCR products and expression vectors were digested with restriction enzymes (New England Biolabs), purified, and ligated using T4 DNA Ligase (New England Biolabs). All constructs were transformed into chemically competent *E. coli* DH5α cells, selected on LB agar plates containing appropriate antibiotics, and verified by Sanger sequencing prior to use.

### Mammalian cell culture

HEK293 cells (human embryonic kidney cell line), HEK293T cells (human embryonic kidney cells expressing SV40 large T antigen), NK92 cells (natural killer cell line), and Molt4 cells (acute lymphoblastic leukemia cell line) were purchased from the American Type Culture Collection (ATCC). HEK293-LFA-1 cells, a derivative of HEK293 cells stably overexpressing lymphocyte function-associated antigen 1 (LFA-1), were generated in-house. HEK293, NK92, and Molt-4 were authenticated with short tandem repeat profiling.

HEK293, HEK293T, and HEK293-LFA-1 cells were cultured in Dulbecco's modified Eagle's medium (DMEM) (Hyclone Laboratories). NK92 and Molt4 cells were cultured in Roswell Park Memorial Institute-1640 (RPMI-1640) medium (Hyclone Laboratories). All media were supplemented with 10% (v/v) heat-inactivated fetal bovine serum (FBS) (Hyclone Laboratories) and 100 IU/ml penicillin and streptomycin (Gibco, Waltham, MA). For NK92 cells, RPMI-1640 medium was further supplemented with 1 mM sodium pyruvate (Gibco), 5% horse serum (Hyclone Laboratories), 2 mM glutamine (Gibco), and 100 IU/ml IL-2 (Peprotech).

NK92 cells stably expressing doxycycline-inducible shRNA targeting kindlin-3 were generated using a lentivirus-based transduction system. Two shRNA sequences targeting kindlin-3 (sh1 K3: 5′-GCCGAATTGTACACGAGTATA-3′ and sh2 K3: 5′-GACTGGTCAGACCATGCTATT-3′) and a control shRNA targeting luciferase (shLuc: 5′-GATTTCGAGTCGTCTTAAT-3′) were synthesized with flanking restriction sites and cloned into the doxycycline-inducible Tet-pLKO-puro lentiviral vector (a gift from Dmitri Wiederschain, Novartis Institutes for BioMedical Research, Cambridge, MA, Addgene plasmid #21915). Lentiviral particles were produced by co-transfecting HEK293T cells with shRNA transfer plasmids, the envelope plasmid pVSVG (a gift from Bob Weinberg, Whitehead Institute for Biomedical Research, Cambridge, MA, Addgene plasmid #8454) and packaging plasmids pMDLg and pRSV-Rev (a gift from Didier Trono, Cell Genesys, Foster City, CA, Addgene plasmid #12251 and #12253) using a standard polyethylenimine (PEI) transfection protocol. 48 h post-transfection, the culture supernatant containing lentiviral particles was used to transduce NK92 cells (1 × 10^6^ cells) using spinoculation in the presence of Retronectin (Takara Bio Inc., Kusatsu, Shiga, Japan) according to the manufacturer's instructions. Transduced NK92 cells were selected with 2 μg/ml puromycin (Thermo Scientific, Waltham, MA) 24 h post-transduction.

Kindlin-3 knockout (KO) Molt4 cells were previously generated in-house using the CRISPR-Cas9n system with the guide RNAs targeting sequences 5′-GGGCTACCGCCAACACTGGG-3′ and 5′-GGGGCCTTCGTGGGATGCTG-3 as reported previously (6) and verified through Western blotting using anti-Kindlin-3 monoclonal antibody (clone 9). Successful gene disruption was further confirmed by Sanger sequencing of the CRISPR-targeted region in the Kindlin-3 gene. A validated Kindlin-3 KO clone was selected for subsequent complementation studies using lentiviral transduction. Complementation constructs included wild-type Kindlin-3 (WT), an integrin-binding-defective mutant (IBD), and a chimeric form in which the F0 subdomain of Kindlin-3 was replaced with that of Kindlin-2 (K2F0K3). Each cDNA was subcloned into the pLenti-CMV-GFP-Zeo (637-7) vector (a gift from Eric Campeau and Paul Kaufman, University of Massachusetts Medical School, Worcester, MA, Addgene plasmid #17449), with a C-terminal P2A self-cleaving peptide sequence to enable bicistronic expression of Kindlin-3 and green fluorescent protein (GFP). Lentiviral particles were produced and used to transduce Kindlin-3 KO Molt4 cells as previously described. GFP-positive cells were analyzed by flow cytometry 24 h post-transduction to confirm transgene expression.

Subsequently, WT Molt4, K3KO Molt4 and complemented Molt4 cells were transduced with IL-2Rβ–mCherry *via* lentiviral delivery, as described above, and mCherry-positive cells were sorted by flow cytometry 24 h later.

### Knockdown induction and cell treatment

For experiments involving NK92 shRNA cells, kindlin-3 knockdown was induced by treating the cells with 1 μg/ml doxycycline for 7 days (doxycycline was replenished every 72 h). Cells were harvested and subjected to immunoblotting to confirm knockdown efficiency. For experiments requiring IL-2 starvation, these NK92 cells were subsequently washed and cultured in complete NK92 growth medium lacking IL-2 for 24 h prior to the assay.

### Immunoprecipitation

Cells (1.5 × 10^7^) were lysed in lysis buffer (10 mM Tris, 100 mM NaCl, 0.5% (v/v) NP-40, pH 7.4) containing protease inhibitor cocktail (Millipore, Burlington, MA) on ice for 30 min 100 μl Gammabind G Sepharose beads (Cytiva) were incubated with 3 μg of relevant serum IgG for 30 min on ice before washing with lysis buffer to remove any unbound serum IgG. The lysate was pre-cleared by rolling with serum IgG–loaded Gammabind G Sepharose beads for 30 min at 4 °C, and the supernatant was subsequently separated into two equal aliquots. Each aliquot was incubated with 6 μg of relevant serum IgG or pull-down antibody, together with 100 μl Gammabind G Sepharose beads. Following incubation on a roller at 4 °C for 3 h, beads were collected and washed thrice with lysis buffer. Bead-bound proteins were eluted by heating for 10 min in 2 × Laemmli buffer (125 mM Tris, pH 6.8, 4% (w/v) SDS, 20% (v/v) glycerol, 0.02% bromophenol blue, 8 M urea) containing 40 mM DTT.

### Immunoblotting

Cells were lysed in ice-cold lysis buffer (25 mM Tris, 150 mM NaCl, 1% (v/v) NP-40, pH 7.4) with protease inhibitor cocktail (Millipore) and phosphatase inhibitor (Nacalai Tesque, Kyoto, Japan). Total protein concentration was determined using a bicinchoninic acid assay kit (Thermo Fisher Scientific) according to the manufacturer’s instructions. Equal protein quantities were separated on 12% SDS–polyacrylamide gels under reducing conditions and subsequently transferred onto PVDF membranes (Bio-Rad Laboratories). PVDF membrane was incubated in Tris-Buffered Saline with Tween-20 (TBST) buffer (25 mM Tris, 150 mM NaCl, 0.1% (v/v) Tween-20) containing either 5% (w/v) bovine serum albumin (BSA) (Sigma-Aldrich) or 5% (w/v) non-fat milk blocking solution at room temperature for 30 min. The membrane was exposed to primary antibodies (Table 1) diluted in blocking solution and rolled at 4 °C overnight. The membrane was washed three times with TBST and then exposed to the relevant HRP-conjugated secondary antibody (Table 2) for 1 h at room temperature. Membranes were washed three times with TBST and then subjected to enhanced chemiluminescence (ECL) detection using an ECL kit (Advansta Inc), and protein bands were captured with a Western blot imaging system (Bio-Rad). Densitometric analysis was performed using ImageJ software (version 1.54).

**Table 1:** Primary antibodies

Antibody	Source	Cat. no	Specificity	Species	Dilution
Kindlin-3 (clone 9)	Generated and purified by TSM lab	NA	Kindlin-3 (F3 subdomain)	Rat	1:1000
IL-2Rβ	Proteintech (Rosemont, IL)	13602-1-AP	IL-2Rβ	Rabbit	1:5000
HA tag	Merck	05–904	HA	Mouse	1:1000
FLAG tag	Proteintech	20543-1-AP	FLAG	Rabbit	1:1000
B-Actin		66009-1-Ig	Beta Actin	Mouse	1:2000
Phospho-p44/42 MAPK (ERK 1/2)	Cell Signaling Technology (Danvers, MA)	9101S	Phospho-p44/42 MAPK (ERK half) (pThr202/pTyr204)	Rabbit	1:1000
p44/42 MAPK (ERK 1/2)		9102S	p44/42 MAPK (ERK half)	Mouse	1:1000
Phospho-Akt		9271S	Phospho-Akt (Ser473)	Rabbit	1:1000
Akt		9272S	Akt	Rabbit	1:1000
Phospho-Stat5		9359S	Phospho-Stat5 (Tyr694)	Rabbit	1:1000
Stat5		94205S	Stat5	Rabbit	1:1000

**Table 2:** Secondary antibodies

Antibody	Source	Cat. no	Dilution
Goat-anti-mouse IgG (H + L) HRP conjugate	Advansta (San Jose, CA)	R-05071–500	1:10,000
Goat-anti-Rabbit IgG (H + L) HRP conjugate		R-05072–500	1:10,000
Goat-anti-rat IgG (H + L) HRP conjugate		R-05075–500	1:10,000

### Protein purification of fusion SUMO-tagged kindlin-1, kindlin-2, and kindlin-3 F0 proteins

Recombinant kindlin-1, -2, and -3 F0 domains were derived from codon-optimized synthetic genes (GenScript, Piscataway, NJ), each fused to an N-terminal cleavable 6 × His-SUMO tag in a pET24a(+) vector, were expressed in *E. coli* BL21(DE3) and purified by immobilized metal affinity chromatography (IMAC). A single transformed colony was inoculated into 50 ml Luria-Bertani (LB) medium containing 50 μg/ml kanamycin and grown overnight at 37 °C at 220 rpm. The overnight culture was then used to inoculate 1 L of fresh LB-kanamycin medium and incubated at 37 °C at 200 rpm until OD_600_ reached 0.6. Protein expression was induced with 0.5 mM isopropyl β-D-1-thiogalactopyranoside (IPTG) and continued overnight at 16 °C at 160 rpm.

The induced culture was harvested by centrifugation at 6000 rpm for 15 min at 4 °C and resuspended in 100 ml of lysis buffer (100 mM HEPES, 150 mM NaCl, 10 mM imidazole, pH 7.4) supplemented with 0.5 mM phenylmethylsulfonyl fluoride (PMSF), 2.5 mM iodoacetamide and protease inhibitor cocktail (Millipore). Lysis was performed by sonication (25% amplitude, 1 s on/off pulses, 20 min), and the lysate clarified by ultracentrifugation at 20,000*g* for 20 min at 4 °C.

The clarified lysate was incubated with 2 ml Ni-NTA beads (Thermo Fisher Scientific) and passed through a chromatography column twice. The column was sequentially washed with 50 ml wash buffer I (20 mM HEPES, 500 mM NaCl, 10 mM imidazole, pH 8.0) and wash buffer II (20 mM HEPES, 500 mM NaCl, 25 mM imidazole, pH 8.0). Bound proteins were eluted with 1 ml elution buffer (20 mM HEPES, 500 mM NaCl, 500 mM imidazole, pH 8.0) in repeated incubations until no protein remained. Eluates were pooled and further purified *via* fast protein liquid chromatography (FPLC) using a Superdex 200 10/300 column (Cytiva) equilibrated in storage buffer (20 mM HEPES, 150 mM NaCl, pH 7.4). Peak fractions were pooled, digested with SUMO protease (Thermo Fisher Scientific) overnight at room temperature, and the cleaved His-SUMO tag was removed *via* reverse IMAC. The flow-through containing tag-free F0 protein was collected, concentrated using Pierce Protein Concentrator (Thermo Fisher Scientific) to a final concentration of 1 mg/ml before use.

### Bio-layer interferometry (BLI)

Binding kinetics were measured using a Gator Prime BLI system (Gator Bio) at 25 °C. All experiments were performed in BLI buffer (20 mM HEPES, 100 mM NaCl, pH 7.4) using black 96-well microplates (Gator Bio) with 200 μl per well and a shaking speed of 1000 rpm.

Streptavidin biosensors were hydrated in BLI buffer for at least 5 min before ligand loading. Biotin-tagged IL-2Rβ peptide or scrambled control peptides were immobilized by dipping the sensors into 600 nM peptide solutions for 300 s, followed by a 120 s wash in buffer. Binding was measured by exposing the sensors to kindlin-1, -2, and -3 F0 domain analytes (0–100 μM) for 300 s (association), then transferring them to buffer for 300 s (dissociation).

To correct for baseline drift, signals from buffer-only reference wells (no analyte) were subtracted. Non-specific binding was accounted for by subtracting responses obtained with scrambled peptides, which do not bind the analyte. Kinetic parameters (association rate constant *k*on, dissociation rate constant *k*off, and equilibrium dissociation constant *K*D) were determined using non-linear regression analysis in GraphPad Prism version 10 (GraphPad Software, La Jolla, CA) based on a 1:1 binding model. Curves were globally fitted across all analyte concentrations to obtain kinetic constants.

### Isotope-labeled protein expression and purification

Human kindlin-3 F0 domain (residues D13-R98) derived from a codon-optimized synthetic gene (GenScript) was cloned as a 6 × His-SUMO fusion construct into the pET24a(+) vector and transformed into *E. coli* BL21(DE3) cells. A single transformed colony was inoculated into 50 ml Luria-Bertani (LB) medium containing 50 μg/ml kanamycin and grown overnight at 37°C at 220 rpm. The overnight culture was used to inoculate 1 L M9 minimal medium supplemented with ^15^N ammonium chloride and ^13^C glucose for isotopic labelling. The culture was incubated at 37 °C at 200 rpm until OD_600_ reached 0.6 to 0.7. Protein expression was induced with 0.5 mM IPTG and continued overnight at 16 °C at 160 rpm.

Cells were harvested by centrifugation at 6000 rpm for 15 min at 4 °C and resuspended in lysis buffer (20 mM HEPES, 500 mM NaCl, 10 mM imidazole, pH 8.0) supplemented with 0.5 mM PMSF and protease inhibitor cocktail (Millipore). The suspension was lysed by sonication (25% amplitude, 1 s on/off pulses, 20 min) and clarified by ultracentrifugation at 20,000*g* for 20 min at 4 °C. The clarified lysate was loaded onto a Ni-NTA gravity column (Thermo Fisher Scientific) equilibrated in lysis buffer. The column was washed with 50 ml wash buffer (20 mM HEPES, 500 mM NaCl, 25 mM imidazole, pH 8.0), and bound protein was eluted with elution buffer (20 mM HEPES, 500 mM NaCl, 300 mM imidazole, pH 8.0). The eluate was buffer-exchanged into lysis buffer using a Superdex 200 10/300 column (Cytiva) by size-exclusion chromatography. The His-SUMO tag was cleaved by incubating the fusion protein with Ulp1 SUMO protease overnight at room temperature. The reaction mixture was passed through a Ni-NTA column to separate the cleaved ^13^C, ^15^N-labeled K3F0 protein (flow-through) from the His-SUMO tag and protease (column-retained). The purified K3F0 was concentrated and further purified by size-exclusion chromatography on a Superdex 200 10/300 column (Cytiva) equilibrated in NMR buffer (20 mM sodium phosphate, 100 mM NaCl, pH 6.5) at a flow rate of 1 ml/min. Peak fractions were pooled and concentrated to 882 μM for NMR analysis.

### NMR spectroscopy and binding analysis

NMR samples containing 882 μM ^13^C, ^15^N-labeled K3F0 protein were prepared in NMR buffer (20 mM sodium phosphate, 100 mM NaCl, 10% (v/v) D_2_O, 1 mM 4,4-dimethyl-4-silapentane-1-sulfonic acid (DSS), pH 6.5). NMR experiments were performed at 298 K on a Bruker DRX 600 MHz spectrometer (Bruker BioSpin) equipped with a cryoprobe. Data were acquired using Bruker Topspin 2.0 software, processed using nmrPipe, and analyzed with CARA software. Three-dimensional heteronuclear NMR experiments (HNCA, HN(CO)CA, HNCACB, and CBCA(CO)NH) were performed for backbone assignment. For binding studies, ^1^H-^15^N HSQC spectra were acquired with 110 μM ^15^N-labeled K3F0 protein in the absence and presence of IL-2Rβ-derived peptide (sequence: RLPLNTDAYLSLQELQGQDPTHLV).

### Flow cytometric analysis of IL-2Rβ internalization

To assess IL-2Rβ internalization, transfected HEK293 or HEK293-LFA-1 cells (2.5 × 10^5^ cells per well) were seeded in 24-well tissue culture plates (Corning) 1 day prior to the assay. On the day of the internalization experiment, cells were washed once with 1 × PBS and incubated with either anti-CD122 antibody (clone TU27, catalog #339002, BioLegend) or mouse IgG isotype control (Sigma-Aldrich) at a final concentration of 2.5 μg/ml, diluted in complete DMEM. The antibody incubation was performed at 4 °C for 30 min.

Following incubation, the antibody-containing medium was removed, and cells were washed once with PBS. IL-2 (Peprotech) was diluted in complete DMEM to a final concentration of 100 IU/ml and added to each well. Cells were then incubated at 37°C to initiate receptor internalization. At the indicated time points (0, 30, 60, and 120 min), cells were harvested using ice-cold 0.5 mM EDTA in PBS and centrifuged at 500*g* at 4 °C for 4 min.

Cells were then incubated with a secondary antibody, FITC-conjugated F(ab′)_2_ fragment of anti-mouse IgG (Sigma-Aldrich), diluted 1:400 in 1 × PBS, for 30 min at 4 °C in the dark. After staining, cells were washed by centrifugation (500*g*, 4 °C, 4 min), resuspended in 1 × PBS, and subjected to flow cytometry using an LSRFortessa X-20 (BD Biosciences). Flow cytometry data were processed and analyzed using FlowJo software version 10.9 (BD Biosciences).

For NK92 experiments, cells were doxycycline-treated as described above prior to IL-2 starvation. On the day of the internalization assay, Fc receptors were blocked by incubating cells with human AB serum (Sigma-Aldrich) at 4 °C for 30 min. All subsequent steps, including primary antibody staining, IL-2 stimulation, harvesting at defined time points, secondary antibody staining, and flow cytometry analysis, were performed as described above for HEK293 cells.

### Flow cytometric analysis of LFA-1 (CD11a/CD18) activation status

Transfected HEK293-LFA-1 cells (2.5 × 10^5^) were seeded in 6 cm tissue culture dishes (Corning) the day before flow cytometry analysis. On the day of the experiment, cells were harvested using 0.5 mM EDTA in PBS. As a positive control for LFA-1 activation, a subset of empty vector-transfected cells was treated with 5 mM MgCl_2_ and 1.5 mM EGTA in complete DMEM at 37 °C for 30 min.

Primary antibodies were diluted in complete DMEM to a final concentration of 2.5 μg/ml: MHM24 (anti-CD11a, clone MHM24, generated and purified in-house), KIM127 (anti-CD18 extended conformation, clone KIM127, generated and purified in-house), and LPM19C (anti-CD11b isotype control, clone LPM19C, generated and purified in-house). Cells were incubated with isotype control or MHM24 antibodies at 4 °C for 30 min, whereas KIM127 antibody incubation was performed at 37 °C for 30 min to preserve the extended integrin conformation.

Following primary antibody incubation, cells were centrifuged at 500*g* at 4 °C for 4 min and stained with FITC-conjugated anti-mouse IgG F(ab′)_2_ fragment (Sigma-Aldrich) diluted 1:400 in 1 × PBS for 30 min at 4 °C in the dark. Cells were washed by centrifugation (500*g*, 4 °C, 4 min), resuspended in 1 × PBS, and analyzed by flow cytometry using an LSRFortessa X-20 (BD Biosciences). Data were analyzed using FlowJo software version 10.9 (BD Biosciences).

For NK92 cells, cells were doxycycline-treated as described above. All subsequent steps, including antibody staining and flow cytometric analysis, were performed as described above for HEK293-LFA-1 cells.

### Surface labeling of IL-2Rβ

Transfected HEK293 or HEK293-LFA-1 cells (3 × 10^6^ cells per plate) were seeded in 6 cm tissue culture dishes (Corning) 1 day prior to the assay. On the day of the assay, cells were washed twice with 1 × PBS and incubated in PBS containing 0.5 mg/ml EZ-Link Sulfo-NHS-Biotin (Thermo Fisher Scientific) for 30 min at 4 °C to label surface proteins. Following incubation, the biotinylation reaction was quenched by adding 3 ml of complete DMEM, and cells were subsequently washed with PBS to remove excess biotin reagent.

Recombinant human IL-2 (Peprotech) was diluted in complete DMEM to a final concentration of 100 IU/ml and added to each plate. Cells were then incubated at 37 °C to initiate receptor internalization. At the indicated time points (0 and 120 min), cells were harvested using ice-cold 0.5 mM EDTA in PBS and centrifuged at 500*g* at 4 °C for 4 min.

Cell pellets were lysed in ice-cold lysis buffer (25 mM Tris, 150 mM NaCl, 1% (v/v) NP-40, pH 7.4) supplemented with a protease inhibitor cocktail (Millipore). Total protein concentrations were quantified using the bicinchoninic acid (BCA) protein assay kit (Thermo Fisher Scientific), according to the manufacturer's instructions. Equal amounts of protein were incubated with streptavidin-conjugated beads (Thermo Fisher Scientific) for 3 h at 4 °C on a rotator to capture biotinylated surface proteins.

After incubation, beads were washed three times with lysis buffer to remove unbound proteins. Bound proteins were eluted by boiling the beads for 10 min in 2 × Laemmli sample buffer supplemented with 40 mM DTT. Eluted proteins were then subjected to SDS-PAGE and Western blotting.

For NK92 experiments, cells were doxycycline-treated as described above prior to IL-2 starvation. All subsequent steps, including surface biotinylation, IL-2 stimulation, cell harvesting, lysis, streptavidin pull-down, and protein elution, were performed as described above for HEK293 cells.

### Analysis of IL-2 sustained signaling

Transfected HEK293 or HEK293-LFA-1 cells (3 × 10^6^ cells per plate) were seeded in 6 cm tissue culture dishes (Corning) 1 day prior to the assay. On the day of the experiment, cells were stimulated with 100 IU/ml recombinant human IL-2 (Peprotech), diluted in complete DMEM, and incubated at 37 °C for 30 min. Following stimulation, the IL-2-containing medium was removed, and cells were washed twice with complete DMEM to terminate stimulation. Cells were then incubated in fresh complete DMEM at 37 °C to assess sustained signaling. At the indicated time points after IL-2 removal (0, 30, 60, and 120 min), cells were harvested by scraping into ice-cold 1 × PBS and processed for downstream immunoblotting (as described above in the Immunoblotting section).

For NK92 experiments, cells were doxycycline-treated as described above prior to IL-2 starvation. All subsequent steps, including IL-2 stimulation (only 25 IU/ml IL-2), washing, and time point harvesting, were performed as described above for HEK293 cells.

### Annexin V/propidium iodide apoptosis detection assay

NK92 cells were doxycycline-treated as described above prior to IL-2 starvation. Following starvation, cells (1 × 10^5^ cells per condition) were cultured in complete NK92 growth medium containing varying concentrations of IL-2 (0, 25, 50, or 100 IU/ml) for an additional 2 days. After treatment, cells were harvested, washed, and stained using the Annexin V Apoptosis Detection Kit (catalogue #556547, BD Biosciences) according to the manufacturer's protocol. Stained cells were analyzed by flow cytometry using an LSRFortessa X-20 (BD Biosciences). Viable cells were identified as Annexin V-negative/PI-negative, early apoptotic cells as Annexin V-positive/PI-negative, and late apoptotic/necrotic cells as Annexin V-positive/PI-positive. Data was processed and analyzed using FlowJo software version 10.9 (BD Biosciences).

### Confocal laser scanning microscopy

8 μm Ni-NTA polystyrene particles (CD Bioparticles) were coated with His-ICAM-1 (Acro Biosystems, Newark, DE) by incubation at room temperature for 30 min. The coated beads (approximately 5 × 10^4^ beads) were then mixed with 50,000 Molt4 cells, followed by the addition of EGTA and MgCl_2_ to final concentrations of 1 mM and 5 mM, respectively, to activate integrins. The mixture was incubated at 37°C for 30 min. Subsequently, the polystyrene particle-cell suspension was seeded onto poly-L-lysine-coated glass-bottom confocal dishes and incubated at 37 °C for 15 min. Cells were fixed for 10 min at RT in 4% (w/v) paraformaldehyde in PBS, permeabilized for 2 min at RT in CSK buffer (100 mM NaCl, 300 mM sucrose, 3 mM MgCl_2_, 1 mM EGTA, 10 mM PIPES, pH 6.8) with 0.25% (v/v) Triton X-100, and washed thrice with PBS.

Non-specific binding was blocked in PBS supplemented with 5% (w/v) bovine serum albumin (BSA) at room temperature for 30 min. Cells were incubated with mouse anti-CD11a antibody (Proteintech) at 1:50 dilution in blocking buffer for 1 h at room temperature, washed with PBS, and incubated with Alexa Fluor 405-conjugated goat anti-mouse IgG (Invitrogen) at 1:150 dilution in blocking buffer for 60 min at room temperature. After a final PBS wash, images were acquired on a Zeiss LSM980 with Airyscan 2 confocal microscope (Carl Zeiss, Oberkochen, Germany) using a 100 × oil-immersion objective lens. Images were processed using Zen 3.3 software (Carl Zeiss) and ImageJ version 1.54f (National Institutes of Health, Bethesda, MD). Colocalization between LFA-1 (Alexa Fluor 405) and IL-2Rβ-mCherry fluorescence at bead contact sites was quantified in ImageJ by calculating the ratio of mCherry signal intensity to Alexa Fluor 405 signal intensity for each analyzed cell-bead conjugate.

### Statistics and reproducibility

GraphPad Prism software version 10 (GraphPad Software) was used for all statistical analyses. Data are presented as mean ± standard deviation (SD) from at least three biological replicates unless otherwise stated. Statistical significance was assessed using one-way ANOVA for single factor experiments or two-way ANOVA for experiments with two independent variables, followed by Dunnett's multiple comparisons test to compare treatment groups against control. A *p*-value < 0.05 was considered statistically significant. Exact *p*-values and statistical test details are indicated in the figure legends.

## Data availability

All data are contained within the manuscript. Source data will be uploaded and accessible *via* the Digital Repository (DR) of Nanyang Technological 790 University (NTU), DR-NTU, Singapore (https://dr.ntu.edu.sg/) upon publication.

## Supporting information

This article contains supporting information.

## Conflict of interest

The authors declare that they have no conflicts of interest with the contents of this article.

## Figures and Tables

**Figure S5:**
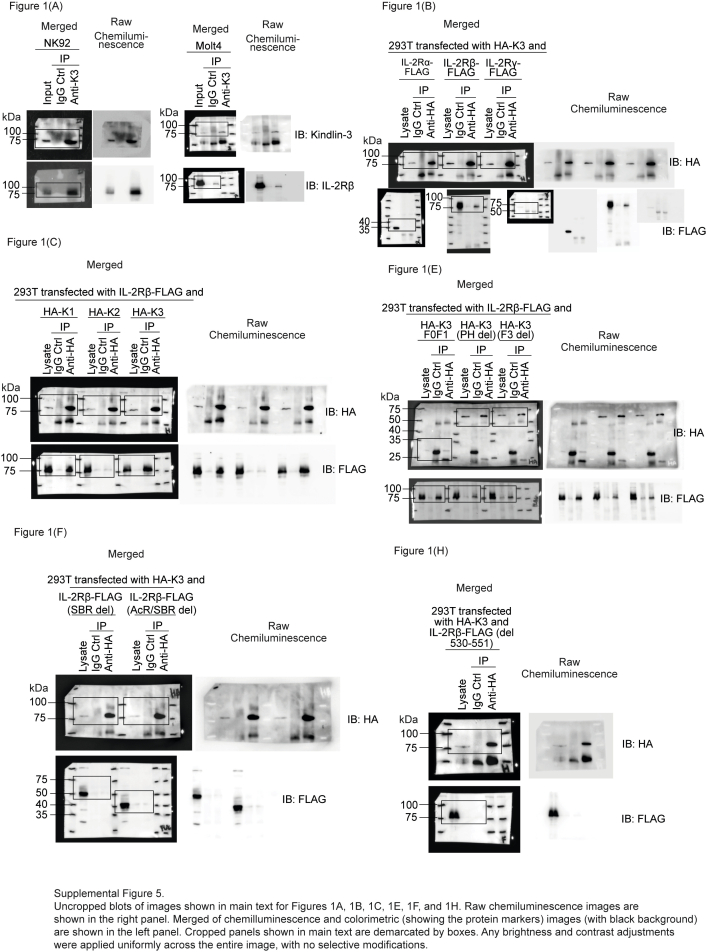


**Figure S6:**
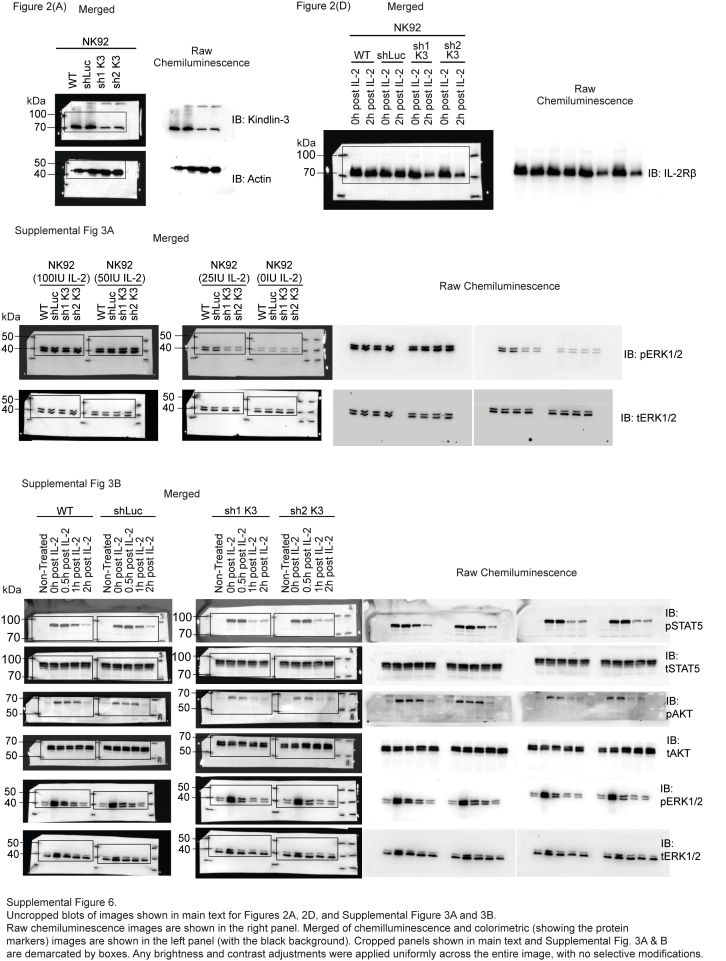


**Figure S7:**
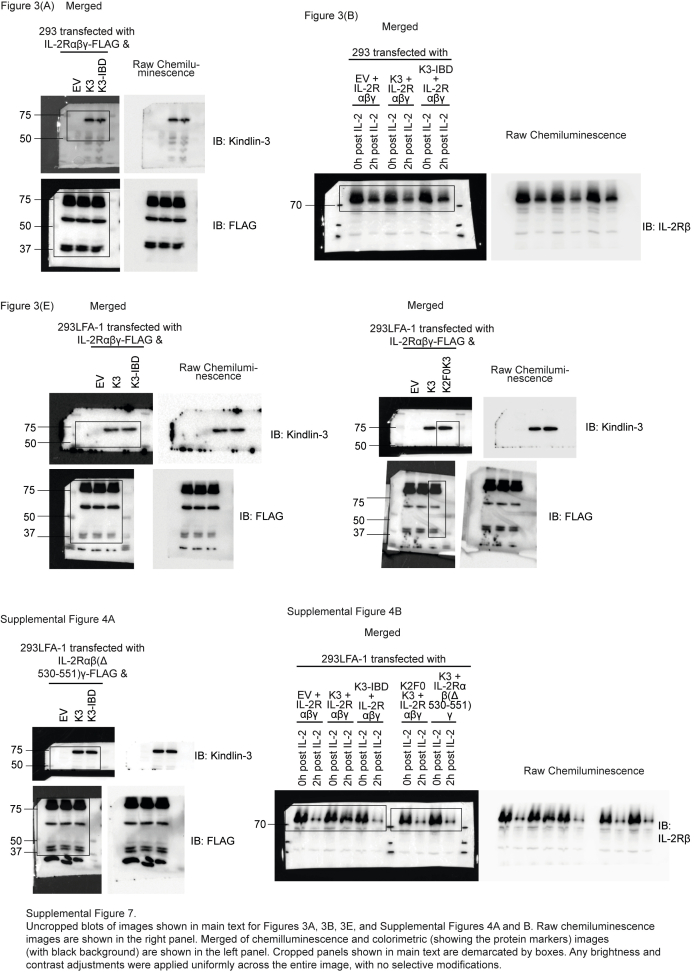


**Figure S8:**
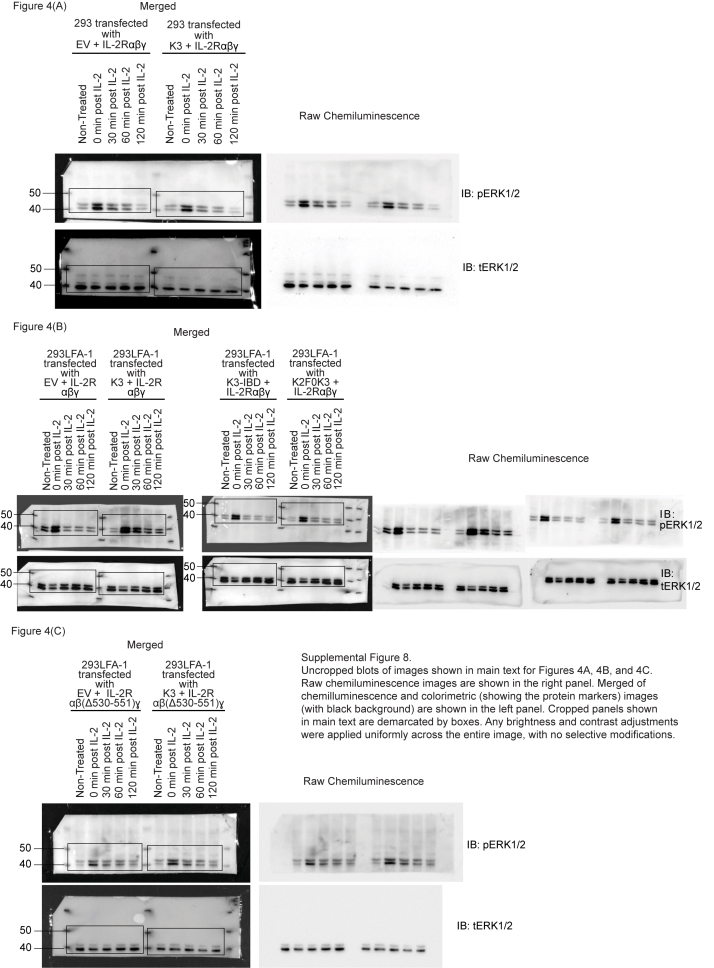


**Figure S9:**